# Systematic review on dental caries preventive and managing strategies among type 2 diabetic patients

**DOI:** 10.3389/froh.2022.998171

**Published:** 2022-11-18

**Authors:** Haoran Chen, Robert Hill, Aylin Baysan

**Affiliations:** Centre for Oral Bioengineering, Institute of Dentistry, Barts and the London School of Medicine and Dentistry, Queen Mary University of London, London, United Kingdom

**Keywords:** dental caries, type-2 diabetes mellitus, management, cariology, minimal invasive approaches

## Abstract

**Purpose:**

The purpose of this systematic review was to evaluate current evidence to prevent and manage dental caries in patients with type 2 diabetes mellitus (T2DM).

**Methods:**

Following Preferred Reporting Items for Systematic Reviews and Meta-Analyses (PRISMA) guidelines, the Participants, Intervention, Comparison, Outcomes and Study Design (PICOS) strategy was used to formulate a structured search: systematic search of PubMed, Cochrane Library, MEDLINE *via* Ovid, EMBASE, Scopus, Web of Science, and Lilacs without any date limit and/or language restrictions. Two independent reviewers performed data extraction and risk of bias assessments in the included studies. Data homogeneity was assessed according to interventions for treating dental caries in T2DM. Statistical analyses were performed with JMP^®^.

**Results:**

Two studies out of 909 were included in the systematic review. Only quantitative studies involving topical applications for management of dental caries in patients with T2DM were included. One study assessed the effect of intensive oral hygiene care program including toothbrushing and interdental cleaning using interproximal brushes and/or dental floss and supragingival debridement by dental hygienist with educational brochures in T2DM, while another investigated the immunologically active salivary substitutes with using Oral Hygiene Instructions (OHI), mouthwash, and moisturizing gel for 6 months. Intensive oral hygiene care program or immunologically active salivary substitutes with using OHI, mouthwash, and moisturizing gel for 6 months were reported to reverse/arrest dental caries in patients with T2DM.

**Conclusion:**

The current randomized controlled clinical trials demonstrated that regular extensive oral health education using interdental cleaning aids, mouthwash, moistening gel, and saliva substitutes including lactoperoxidase, lysozyme, glucose oxidase, and lactoferrin could control oral inflammation and contribute to the management of dental caries in patients with T2DM.

**Systematic Review Registration:**

https://www.crd.york.ac.uk/prospero/display_record.php?ID=CRD42020197507, identifier: CRD42020197507.

## Introduction

Diabetes mellitus (DM) is considered the most common worldwide health issue affecting almost 1 in 11 adults. The prevalence of this disease for all-aged groups worldwide was 2.8% in year 2000 and is estimated to reach 4.4% by 2030 (1). The global mortality for DM was reported to be approximately 2.9 million and to account for 5.2% of all deaths by 2000 (2). The International Diabetes Federation reported that type 2 diabetes mellitus (T2DM) caused five million deaths in the age of 20–79 years in 2015 (3). Interestingly, 90%–95% of the DM cases are patients with T2DM (4, 5).

The reported oral health complications related to T2DM are periodontal diseases, salivary dysfunction, dental caries, odontogenic abscesses, tooth loss, soft tissue lesions of the tongue and mucosa, candidiasis, and taste (6, 7). Almusawi et al. investigated the potential risk factors of dental caries in patients with T2DM. Dental caries risk could be related to the fasting glucose blood, hemoglobin A1c (HbA1c), and salivary glucose (8). However, there is sparse evidence to support a potential association between T2DM and dental caries (9, 10). As a consequence, DM and related oral complications with a sequela of events, i.e., xerostomia, periodontal diseases with exposed root surfaces, form a vicious cycle resulting in compromised wellbeing and quality of life for patients with T2DM.

Holistic patient care is based on early detection of dental diseases, and preservation of hard and soft tissues (11–15). Early caries detection monitors progression of dental carious lesions, which contributes to formulating personalized management plans and preventing further carious lesion activity. In addition, tailored patient care approaches aim to control the caries progression rather than simple removal of carious lesions and restoration (“drilling and filling”) (12).

In this respect, implementation of personalized care strategies in clinical practice would manage oral complications of patients with T2DM and prevent tooth loss. However, there is lack of standard operating procedures for the management of dental caries in patients with T2DM. Therefore, this systematic review aimed to systematically assess the available clinical evidence for the management of dental caries using minimally invasive (MI) strategies in patients with T2DM.

## Materials and methods

### Study registration

The study protocol was registered in PROSPERO database (Registration ID CRD42020197507). This systematic review was conducted in accordance with the Cochrane handbook guidelines for systematic review of interventions (16). The study methodology followed the four-phase diagram of the Preferred Reporting Items for Systematic Reviews and Meta-Analyses (PRISMA) (17).

### Search, eligibility criteria, and study selection

The search strategy used three concepts: first concept included any words related to dental caries while the second concept involved T2DM. Finally, the third concept incorporated either topical applications with patient education or conventional treatment “drilling and filling”.

The Participants, Intervention, Comparison, Outcomes and Study Design (PICOS) (Table 1) was used to formulate an effective search strategy by defining the selected criteria based on participants, interventions, comparisons, and outcomes. Participants were adults (>18 years of age) of any ethnic group who were diagnosed with T2DM. The interventions included patient education and oral hygiene education, using toothpaste containing different concentrations of fluoride alone or with the addition of remineralizing or antibacterial agents, fluoridated mouth rinse, silver diamine fluoride (SDF) solution, fluoride gel, dental varnish containing fluoride/CPP-ACP, chlorhexidine, ozone, polyol, and probiotics. The ultimate outcome was the arrestment/reversal of dental caries. The study design included quantitative, randomized controlled clinical trials, nonrandomized controlled trial (RCT), cross-sectional, prospective, and retrospective studies. Furthermore, systematic reviews and meta-analysis, review articles, observational studies, case reports, expert opinions, and laboratory-based studies were excluded. A comprehensive search was conducted to identify potentially relevant studies by exploring a range of electronic databases (PubMed, Cochrane Library, MEDLINE *via* Ovid, EMBASE, Scopus, Science Direct, Web of Science Core Collection, and Lilacs). Additionally, Google Scholar search for references were undertaken to identify any other relevant published work. The search was carried out without applying any time limits or language restrictions until May 2020. The full search strategy is described in Table 2.

**Table 1:** PICOS research question development.

		Inclusion criteria	Exclusion criteria
*P*—Participant	Participants ≥ 18 with DM type 2 and dental caries		Participants with DM type 1 Participants < 18 years old
I—Intervention	Management of dental caries using topical applications with patient education and oral hygiene education, different concentration fluoride toothpaste alone or with the addition of remineralizing or antibacterial composition, fluoridated mouth rinse, silver diamine fluoride (SDF), fluoride gel, fluoride varnish or CPP-ACP, chlorhexidine, ozone, polyol, and probiotics	
C—Comparison	Conventional treatment “drilling and filling”	
O—Outcome	Arrest/reversal of dental caries	Mean numbers of dental carious lesions that have been arrest/reversal
		Proportion of dental carious lesions that have been arrest/reversal
		No symptoms of pain or discomfort
	Restorations	Survival of restorations
S—Study design	Quantitative studies, randomized controlled clinical trials (RCTs), non-RCTs, cross-sectional studies, *p*rospective studies, and retrospective studies		Systematic reviews and meta-analysis, review articles, observational studies, case reports, expert opinions, laboratory study, laboratory-based, *in vitro* studies, animal studies, and studies using extracted permanent teeth

DM, diabetes mellitus.

**Table 2:** Full search strategy in database.

#1	“dental caries” OR “carious lesion” OR “caries lesion” OR “enamel caries” OR “root caries” OR “coronal caries” OR “cariogenesis” OR “carious dentine” OR “carious teeth” OR “dental caries susceptibility” OR “dental decay” OR “tooth caries” OR “tooth decay” OR “proximal caries” OR “interproximal caries” OR “occlusal caries” OR “initial caries lesion” OR “primary caries” OR “secondary caries” OR “recurrent caries” OR “residual caries” OR “hidden caries” OR “rampant caries” OR “dental caries”[MeSH] OR “root caries”[MeSH]
#2	“diabetes mellitus, type 2”[MeSH] OR “diabetes mellitus type 2” OR “type 2 diabetes mellitus” OR “diabetes mellitus”[MeSH] OR “diabetes mellitus” OR “diabetes”
#3	“oral hygiene education” OR “fluoridate” OR “fluoridation”[MeSH] OR “fluoridation” OR “fluorides”[MeSH] OR “fluorides” OR “fluoride” OR “topical fluoride” OR “topical fluorides” OR “fluorides, topical”[MeSH] OR “toothpastes”[MeSH] OR “toothpastes” OR “toothpaste” OR “dentifrices”[ Pharmacological Action] OR “dentifrices”[MeSH] OR “dentifrices” OR “dentifrice” OR “cariostatic agents” OR “cariostatic agents”[MeSH] OR “cariostatic agents”[Pharmacological Action] OR “mouthwashes”[MeSH] OR “mouthwashes” OR “silver diamine fluoride” OR “silver diamine fluoride”[Supplementary Concept] OR “silver diammine fluoride” OR “SDF” OR “gel” OR “dental varnish” OR “casein phosphopeptide-amorphous calcium phosphate” OR “CPP-ACP” OR “chlorhexidine”[MeSH] OR “chlorhexidine” OR “chlorhexidin” OR “ozone”[MeSH] OR “ozone” OR “polyol”[Supplementary Concept] OR “polyol” OR “polyoles” OR “polyols” OR “sugar alcohols” OR “sugar alcohols”[MeSH] OR “probiotics”[MeSH] OR “probiotics” OR “probiotic” OR “lasers”[MeSH] OR “lasers” OR “laser” OR “lesion exposure” OR “infiltrate” OR “infiltrated” OR “infiltrates” OR “infiltration” OR “infiltrations” OR “atraumatic restorative treatment” OR “atraumatic” OR “restoration” OR “restorations” OR “restorable” OR “restorated” OR “restorative” OR “restoratives” OR “restore” OR “restored” OR “restores” OR “restoring” OR “minimally invasive dentistry” OR “MID” OR “MI” OR “minimally invasive” OR “minimally invasive surgical procedures”[MeSH] OR “plaque control” OR “varnish” OR “varnished” OR “varnishing” OR “varnishes” OR “paint”[MeSH] OR “paint” OR “sealant” OR “sealants” OR “triclosan”[MeSH] OR “triclosan” OR “essential oils” OR “thyme” OR “thymus plant”[MeSH] OR “thymus plant” OR “thymus” OR “remineralisation” OR “remineralise” OR “remineralised” OR “remineralising” OR “remineralization” OR “remineralize” OR “remineralized” OR “remineralizing” OR “demineralisation” OR “demineralise” OR “demineralised” OR “demineralising” OR “demineralization” OR “demineralizations” OR “demineralize” OR “demineralized” OR “demineralizes” OR “demineralizing” OR drill, fill OR “conventional treatment”
#4	Search #1 AND #2 AND #3.

Boolean operators (“OR” and “AND”) were used to join search terms.

### Data collection

Duplicate articles were removed using the EndNote X9 software (Clarivate Analytics, Philadelphia, Pa, United States) (18). The screening and data extraction processes were performed with the Covidence web-based software (Melbourne, Victoria, Australia)^1^. Based on the selection criteria through PICOS strategy, titles and abstracts were examined independently by two examiners (HC and AB), and any disagreements were resolved according to a predefined strategy, using consensus and arbitration as appropriate. The second stage consisted of reading the full texts and assessing the potential studies thoroughly. Studies not meeting the inclusion criteria were removed. If a disagreement could not be resolved, then a third investigator (RH) was approached to reach the consensus. The references cited in the included studies were also checked.

### Risk of individual bias of the studies

Two reviewers independently assessed the risk of bias according to the Cochrane library guidelines (19), and any disagreement was resolved with the third reviewer (RH). Five domains were identified for risk of bias: selection bias, performance bias, detection bias, incomplete data, and reporting bias. Subsequently, each domain judgment was marked for each study as low risk of bias, high risk of bias, or unclear.

### Syntheses of results

Data homogeneity was assessed according to the management of dental caries in patients with T2DM. The significant level was set at 0.05, using JMP®, Version 14.2 (SAS Institute, United States). The variables related to study design, location, duration, dropout rate, sample size, age of participants, gender, dental caries diagnostic criteria, evaluation methods, intervention methods used, outcome measure, and results were recorded.

## Results

**Figure 1:**
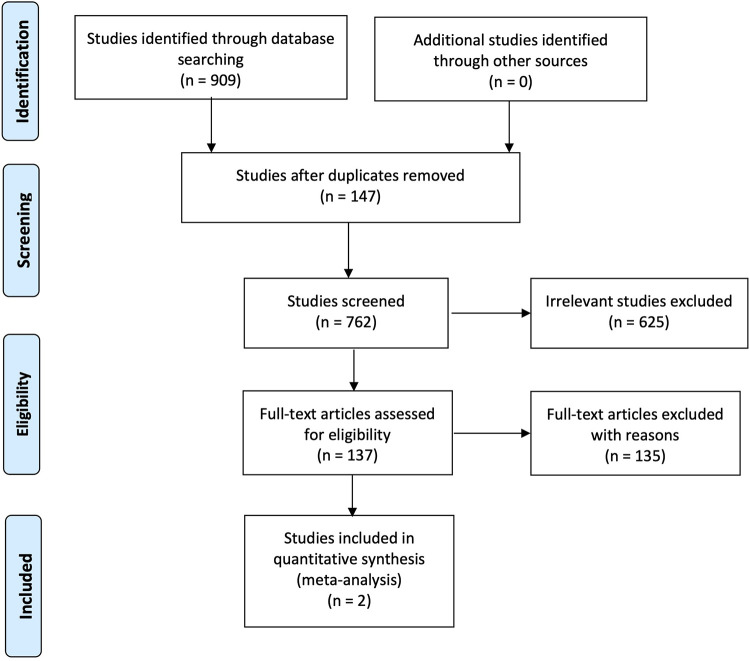
PRISMA flow diagram.

The search methodology has been reported according to the PRISMA Statement and presented in Figure 1. A total of 909 studies were initially identified in all searched database. 147 studies were then excluded as duplicates. The titles and abstract were examined according to inclusion criteria, and 625 articles were subsequently excluded leaving 137 studies for further review. Search strategy used in PubMed (MEDLINE) (*n* = 75), EMBASE (*n* = 123), Ovid (MEDLINE) (*n* = 15), Web of Science (*n* = 217), Scopus (*n* = 479), Cochrane Library (*n* = 0), and Lilacs (*n* = 0) were identified.

Following the assessment of these studies, 135 studies were then excluded, and the reason is that 41 studies presented non-T2DM participants. These studies recruited healthy participants (*n* = 4), patients requiring orthodontic treatments (*n* = 1), nursing home residents (*n* = 2), patients with renal disease (*n* = 1), institutionalized elderly people (*n* = 2), diabetes type 1 participants (*n* = 3), patients with odontogenic infections (*n* = 1), participants with nasopharyngeal carcinoma (*n* = 1), head-and-neck cancer patients (*n* = 9), Sjogren's syndrome (*n* = 5), oral cancer (*n* = 1), xerostomia patients (*n* = 7), participants with lower-arch intra-oral appliances (*n* = 1), hematopoietic stem cell transplantation patients (*n* = 1), independently living older adults (*n* = 1), and finally patients with metabolic syndrome (*n* = 1).

Four studies failed to specify the type of diabetes, while background information of participants was missing in seven studies. In addition, the reported outcomes in two studies were not related to dental caries.

Finally, 23 studies were excluded due to different study designs. Three studies were systematic reviews while 8 of 23 were laboratory-based studies. There were 44 studies with noninterventional designs. These were cross-sectional (*n* = 33), case–control (*n* = 6), survey (*n* = 1), and observational (*n* = 4) studies. There was one unpublished data while the authors from two studies failed to reply to the request for more information with regard to methodology.

A total of only two studies fulfilled the criteria specified in this systematic review. The key characteristics of included studies are shown in Table 3. Both studies that evaluated dental caries using the MI approach in patients with T2DM were randomized controlled trials. One study was based on Korea recruited participants from university hospital (20), while the other study was conducted with the participants from the Local Health Unit in Italy (6). These two studies examined the changes in dental caries status for patients with T2DM. The sample size varied from 75 to 134 participants and the period of these studies was 6 months with follow-up rates between 93.75% and 100%, respectively (Table 4).

**Table 3:** Reported key characteristics of included studies.

Authors	Methods	Participants	Study groups	Outcome measures
Lee et al. (2009)	**Location:** Department of internal medicine, Yeungnam University hospital, Daegu City **Follow-up:** 6 months **Funding source:** University grants **Study design:** Randomized controlled trial	***N* =** 75 Type-2 diabetes (28 F and 47 M) **Age:** < 45 (*n* = 20) 45–54 (*n* = 21) 55–64 (*n* = 25) >65 (*n* = 9)	**Intervention group:** Intensive oral hygiene care program once a month for 6 months (*n* = 40) **Control group:** Intensive oral hygiene care program at baseline and after 6 months only (*n* = 35)	Dental caries status
Montaldo et al. (2010)	**Location:** Local health unit “Napoli 1”, Italy **Follow-up:** 6 months **Funding source:** Not reported **Study design:** Randomized controlled trial	***N* =** 134 type 2 diabetes (78 F and 56 M) **Age:** 47.9 ± 2.9 years	**Intervention group:** Immunologically active salivary substitutes with toothpaste, mouthwash, and a moistening gel (*n* = 67) **Control group:** No intervention (*n* = 67)	Dental caries status

**Table 4:** Comparison of included studies.

Study	Evaluation methods	Results	Dropout rate
Lee et al. (2009)	Clinical assessments and radiographs	**Baseline values (mean ± SD) for intervention and control groups, respectively** DT index (1.28 ± 2.90; 1.60 ± 0.91) (*p *= 0.51), DMFT index (6.98 ± 5.20; 8.77 ± 3.99) (*p* = 0.10), Plaque index (7.38 ± 4.05; 11.14 ± 3.72) (*p* < 0.0001), Dental calculus index (9.75 ± 3.10; 9.34 ± 2.48) (*p* = 0.53), Bleeding index (1.95 ± 0.75; 2.11 ± 0.54) (*p *= 0.10), PHP index (3.25 ± 0.94; 4.01 ± 0.70) (*p* = 0.0002), HbA1c (7.9% ± 1.9%; 7.5% ± 1.2%) (*p* = 0.32) **Values at 6 months (mean ± SD) for intervention and control groups, respectively** DT index (1.33 ± 2.89; 1.57 ± 0.92) (*p *= 0.10), DMFT index (7.18 ± 5.22; 8.86 ± 4.04) (*p* = 0.40), Plaque index (1.83 ± 0.90; 9.83 ± 4.05) (*p* < 0.0001), Dental calculus index (1.35 ± 0.74; 8.06 ± 2.83) (*p* < 0.0001), Bleeding index (0.32 ± 0.33; 1.92 ± 0.68) (*p* < 0.0001), PHP index (1.46 ± 0.45; 3.96 ± 0.85) (*p* < 0.0001)	6.25%
Montaldo et al. (2010)	Clinical assessments	**Values for intervention group at baseline and after 6 months** Patients with carious or restored teeth ≤ 2 [32 (48%) to 33 (49%)] (*p *> 0.01), Patients with carious or filled teeth ≥ 3 [28 (42%) to 29 (43%)] (*p *> 0.01), Dental Plaque index (2.3 ± 0.73 to 1.6 ± 0.56) (*p *< 0.01), Positive yeast counts [40 (60%) to 25 (37%)] (*p *< 0.01) **Values for control group at baseline and after 6 months** Patients with carious or restored teeth ≤ 2 [33 (49%) to 34 (51%)] (*p *> 0.01), Patients with carious or filled teeth ≥ 3 [26 (39%) to 28 (42%)] (*p *> 0.01), Dental Plaque index (2.1 ± 0.74 to 2.2 ± 0.71) (*p* > 0.01), Positive yeast count [38 (57%) to 41 (61%)] (*p* > 0.01)	35.3%

SD, standard deviation; DT, Decayed Teeth; DMFT, Decayed, Missing, and Filled Teeth; PHP, Patient Hygiene Performance; Hb1Ac, hemoglobin A1c.

Lee et al. carried out oral health education that included instructions on toothbrushing and the use of oral health aids (interproximal brushes and/or dental floss) from a trained dental hygienist in addition to educational brochures. The hygienist then performed supragingival debridement under the supervision of a trained dentist. All these procedures (oral examination, full-mouth radiograph, oral health education, and supragingival debridement) were repeated on a monthly basis for the intervention group. However, the control group had all these procedures at baseline and after six months only (20). The study concluded that plaque, dental calculus, bleeding, and Patient Hygiene Performance (PHP) indices reduced when compared to baseline values in both groups. However, there were significant differences in plaque, dental calculus, bleeding, and PHP indices (*p *< 0.001) between the intervention and control groups after 6 months. The number of Decayed Teeth (DT) index showed a decrease in the number of dental caries for a period of 6 months in the control group. Interestingly, this index increased in the intervention group (*p *> 0.05). The Decayed, Missing, and Filled Teeth (DMFT) indices were increased in both groups after 6 months. However, the DMFT index failed to show any significant differences between the intervention and control groups (*p *> 0.05). The mean level of HbA1c was 7.9 ± 1.9 and 7.5 ± 1.2 in the intervention and control groups, respectively, at the baseline (*p *> 0.05). It should be noted that this study only provided HbA1c status for both groups at baseline (20).

Montaldo et al. applied immunologically active salivary substitutes including lactoperoxidase, lysozyme, glucose oxidase, and lactoferrin with oral hygiene instructions (including the use of toothpaste, mouthwash, and moisturizing gel) to assess the participants with T2DM either having dental caries or restorations and compared to the control group (no intervention) (6). In this 6-month study, there was 1% increase in the intervention group for patients with ≥3 carious or restored teeth when compared to the control group with 3% increase (*p *> 0.01). The percentage of number of carious or restored teeth ≤2 increased 1% in interventional group, while the increase was 2% in the control group (*p *> 0.01). In addition, mean differences in dental plaque index for the intervention group was 0.7 (*p *< 0.01); however, this difference for the control group was 0.1 (*p *> 0.01). There were no statistical differences in dental caries and tooth loss between the intervention and control groups (*p *> 0.01). In addition, microbiological analysis showed that the salivary substitutes significantly decreased the yeast counts (*Candida albicans*, *Candida tropicalis*, and *Candida* and *Aspergillus* species) in the intervention group (*p* < 0.01); however, the yeast counts increased in the control group (*p *> 0.01) (6).

The Inter-Examiner Agreement was also observed with two studies (Table 5). There was an unclear risk of selection bias as the participants were randomly allocated to two groups according to the order of presentation at the outpatient clinics; however, there were no details in the randomization process (20). The recruitment and randomization processes were also vague in the study conducted by Montaldo et al. Therefore, the risk of selection bias was considered to be unclear (6).

**Table 5:** Risk of bias in the included studies.

	Lee et al. (2009)	Montaldo et al. (2010)
Selection bias (sequence generation, allocation concealment)	?	?
Performance bias (blinding of participants)	H	L
Detection bias (blinding of outcome assessment)	H	L
Incomplete data	H	L
Reporting bias (selective reporting)	L	L

? Unknown risk of bias; H, high risk of bias; L, low risk of bias.

Montaldo et al. had the same clinicians to carry out the dental examination at baseline and after 6 months. The clinicians were blinded to the intervention and control groups. Therefore, the performance and detection biases were considered to be low (6). Lee et al. required the clinicians to be blinded *prior* to all clinical assessments at baseline only; however, there was no clearly defined blinding process. Therefore, this might affect the clinicians to conduct clinical examinations and oral health education for interventional and control groups. The performance and detection biases are likely to be high in this study (20).

Regarding the incomplete data, there was low risk in two studies. Lee et al. lost five participants who were in the control group (80 patients were included in each group at the baseline) after 6 months. There were no detailed reasons such as time points for the lost participants in the study. The risk bias for incomplete data is likely to be high (20). There were no dropouts after 6 months in the study by Montaldo et al. The bias of incomplete data is considered to be low (6).

Overall, the risk of reporting bias in two studies is likely to be low since the measured outcomes were fully reported.

## Discussion

In this systematic review, the clinical outcome was defined as arrest/reversal of dental caries. In the included studies, the primary outcome was periodontal health (20) with dental caries (6) in patients with T2DM. These authors evaluated dental caries status following the employment of oral health education and different minimally invasive interventions. This is the first systematic review to identify the necessity of oral health education and minimally invasive strategies for the management of dental caries in patients with T2DM.

T2DM is one of the risk factors for hyposalivation and this might be related to the structural changes caused by diabetes mellitus in the salivary glands, changes consisting of acinar atrophy, and adipose infiltration. In addition, the quality of saliva might also be affected due to the oral complications caused by diabetes. Therefore, early detection and management of dental caries in diabetes patients would provide retention of their teeth and improve quality of life. In this respect, topical applications with patient education aim to minimize invasive interventions such as “drilling and filling,” which is not the preferred treatment modality by many patients (21). In this systematic review, topical applications with patient education included oral hygiene instructions and immunologically active salivary substitutes. These management strategies can reduce the progression of dental caries to a certain extent.

Both studies in this systematic review used oral hygiene instructions, which were previously proven to be effective in improving oral health. Nyvad and Fejerskov reported that active root carious lesions can be reversed to inactive status with oral hygiene instructions alone (toothbrushing with fluoridated toothpaste) for a period of 18 months. Therefore, oral hygiene education could be considered as part of patient education and would be beneficial for the management of dental caries in T2DM (22).

As a separate note, Montaldo et al. (6) reported that the reduction in saliva would result in the compromised host-defense mechanism in the mouth due to the possible reduction or lack of salivary proteins and enzymes. Interestingly, the authors also noted that the use of salivary substitutes including lactoperoxidase, lysozyme, glucose oxidase, and lactoferrin could reduce plaque accumulation and indirectly eliminate the risk of gingival inflammation and mucosal infections. In their study, a total of 90 patients out of 134 had gingivitis (67%) and 78 patients out of 134 (58%) had positive yeast counts (*Candida albicans*). Following the use of salivary substitutes, positive yeast counts decreased from 60% to 37%. However, the findings of this study (6) failed to distinguish the effectiveness of immunologically active salivary substitutes and oral hygiene instructions in the reversal/arrest of dental caries for patients with T2DM. The effect of immunologically active salivary substitutes and/or oral health education for the management of dental caries in patients with T2DM requires further exploration.

High levels of salivary glucose can increase the candidal adherence to buccal epithelial cells. Salivary glucose forms chemically reversible glycosylation products with proteins in the oral tissues during hyperglycemic episodes in T2DM patients. This results in the accumulation of advanced glycosylation end products (AGEs). These AGEs would increase the number of receptors for *Candida* species in oral mucosa. In this respect, *Candida* species colonize on teeth and oral mucosa membranes since glucose is a source of nutrient for these microorganisms and ultimately would suppress the killing capacity of neutrophils. Salivary immunoglobulin A and free secretory component are capable of inhibiting the adherence of *Candida* species to the epithelial cells. Therefore, the immunologically active saliva substitutes in T2DM patients could be an effective way to reduce salivary glucose concentration (23) and ultimately reduce the incidence of oral infections and dental caries. However, more evidence is required.

Lee et al. (20) reported an increase in DMFT indices for both groups following the intensive oral hygiene program with educational leaflets from a trained dental hygienist for a period of 6 months. Interestingly, the DT index showed the reduction in dental caries for the control group when the participants had oral hygiene education only twice during the study. However, the interventional group had increase in the DT index for a period of 6 months despite having the oral hygiene instructions on a monthly basis. In addition, the HbA1c levels in the intervention group were worse when compared to those in the control group, which might affect the impact of oral hygiene care in these participants. Yonekura et al. reported that DT was higher in patients with poorly controlled T2DM (24). With this respect, Al Amri et al. indicated that the level of HbA1c was reduced by the implementation of oral hygiene instructions for a period of 2 years. Therefore, it should be noted that education on plaque control might have a potential impact to influence the glycemic control (25, 26). However, patients with T2DM and unstable HbA1c might have more glucose in saliva and could experience dry mouth (27). These conditions then contribute to the increase in biofilm on teeth, which leads to dental caries (28). The level of metabolic control in T2DM could, therefore, affect the management of dental caries (23).

The dental plaque index is used to measure the oral hygiene status (29). Removal of dental plaque is considered a strategy to control dental caries due to the presence of a community of microorganisms that are dominated by acidogenic and antitolerating species within the biofilm (30). In this respect, Von der Fehr et al. reported that high sugar exposure related to the lack of plaque control caused carious lesions during a 23-day period (31). Interestingly, Holmen et al. also concluded that mechanical plaque removal or interruption of biofilm without the use of fluoride could be capable of arresting early enamel caries (32). However, this study was carried out with only four premolars. In addition, dental biofilm removal by toothbrushing with sufficient fluoridated toothpastes on a daily basis contributed to arresting the progression of dental caries in noncavitated and cavitated dentine lesions. However, even after performing dental plaque removal, the residual biofilms and microorganisms could be retained in the surface irregularities and areas such as approximal surfaces and fissures. Therefore, this is unlikely to prevent the development of dental caries.

The DMFT index is one of the most commonly used indices in epidemiologic surveys of dental caries. This index quantifies the dental health status based on the sum of number of decayed, missing due to caries, and filled teeth in the permanent teeth. However, the DMFT index fails to distinguish between decayed, missing, and filled teeth due to an equal weight to the decayed and restored teeth (33). The percentage of carious or restored teeth also failed to distinguish carious and restored teeth in the included study (6). In addition, this system is unable to report noncavitated enamel carious lesions (34). Many authors have proposed different methods to evaluate dental caries, such as the Nyvad criteria (35) and International Caries Detection and Assessment System (ICDAS) (36). These methods have been validated to present reproducibility and correlation in dental caries assessment (37).

One possible limitation in this systematic review is that none of the reviewed publications had comparable figures that would allow for an additional analysis directly to compare their results. The included studies reported completely different measurements such as the DT, missing teeth (MT), filled teeth (FT), DMFT, calculus, plaque, and bleeding indices. In this respect, these values were at different scales. Lee et al. reported the DMFT index to indicate the status of dental caries. However, Montaldo et al. indicated the number of patients with carious/filled and missing teeth in percentages. In addition, Lee et al. presented the mean and standard deviation (SD) for the plaque index, while Montaldo et al. reported the mean of plaque index without specified SD or standard error (SE) (6).

The evidence obtained from this systematic review emphasizes the need for further well-designed, randomized, and controlled clinical trials evaluating the effect of different strategies for management of dental caries in patients with T2DM for a period of at least 2 years.

## Conclusion

In conclusion, the current randomized controlled clinical trials demonstrated the following:

(i) Regular extensive oral health education using interdental cleaning aids, mouthwash, and moistening gel can help in dental caries management and prevention.
(ii) Saliva substitutes including lactoperoxidase, lysozyme, glucose oxidase, and lactoferrin could control oral inflammation and contribute to the management of dental caries in patients with T2DM.

In the future, more randomized controlled clinical trials including prevention and management strategies are required to carry out dental caries treatment in patients with T2DM.

## Data Availability

The original contributions presented in the study are included in the article/Supplementary Material, further inquiries can be directed to the corresponding author.

## Footnotes

1 https://www.covidence.org/.
